# Nanomedicine: A Promising Way to Manage Alzheimer’s Disease

**DOI:** 10.3389/fbioe.2021.630055

**Published:** 2021-04-09

**Authors:** Nazeer Hussain Khan, Maria Mir, Ebenezeri Erasto Ngowi, Ujala Zafar, Muhammad Mahtab Aslam Khan Khakwani, Saadullah Khattak, Yuan-Kun Zhai, En-She Jiang, Meng Zheng, Shao-Feng Duan, Jian-She Wei, Dong-Dong Wu, Xin-Ying Ji

**Affiliations:** ^1^Henan International Joint Laboratory for Nuclear Protein Regulation, School of Basic Medical Sciences, Henan University, Kaifeng, China; ^2^Department of Pharmacy, Faculty of Biological Sciences, Quaid-i-Azam University, Islamabad, Pakistan; ^3^Department of Biological Sciences, Faculty of Sciences, Dar es Salaam University College of Education, Dar es Salaam, Tanzania; ^4^School of Natural Sciences, National University of Sciences and Technology, Islamabad, Pakistan; ^5^School of Stomatology, Henan University, Kaifeng, China; ^6^Institutes of Nursing and Health, School of Nursing and Health, Henan University, Kaifeng, China; ^7^International Joint Center for Biomedical Innovation, School of Life Sciences, Henan University, Kaifeng, China; ^8^Institute for Innovative Drug Design and Evaluation, School of Pharmacy, Henan University, Kaifeng, China; ^9^Brain Research Laboratory, School of Life Sciences, Henan University, Kaifeng, China; ^10^Kaifeng Key Laboratory of Infection and Biological Safety, School of Basic Medical Sciences, Henan University, Kaifeng, China

**Keywords:** Alzheheimer’s disease, pathogenesis, blood brain barrier, nanomedicines, cellular transport, nanoparticles, drug delivery, theranostic

## Abstract

Alzheimer’s disease (AD) is a devastating disease of the aging population characterized by the progressive and slow brain decay due to the formation of extracellular plaques in the hippocampus. AD cells encompass tangles of twisted strands of aggregated microtubule binding proteins surrounded by plaques. Delivering corresponding drugs in the brain to deal with these clinical pathologies, we face a naturally built strong, protective barrier between circulating blood and brain cells called the blood–brain barrier (BBB). Nanomedicines provide state-of-the-art alternative approaches to overcome the challenges in drug transport across the BBB. The current review presents the advances in the roles of nanomedicines in both the diagnosis and treatment of AD. We intend to provide an overview of how nanotechnology has revolutionized the approaches used to manage AD and highlight the current key bottlenecks and future perspective in this field. Furthermore, the emerging nanomedicines for managing brain diseases like AD could promote the booming growth of research and their clinical availability.

## Introduction

Alzheimer’s disease (AD) is a devastating condition of the aging population characterized by the progressive and slow brain decay due to plaque formation in the hippocampus (Querfurth and LaFerla, 2010). It has been found that the formation of those plaques starts up about 20 years before the onset of clinical symptoms, which makes the exact trajectory of pathologies associated with AD unclear (Jack and Holtzman, 2013; Fagan et al., 2014). The incidence of AD is rising worldwide. More than 50 million people were affected with AD in 2019, and its burden is continuously rising, which may lead to an enormous effect on both the world economy and manpower (Patterson, 2018). It is estimated that by the middle of the current century, 13.8 million Americans of age 65 and older may have AD symptoms. AD is the sixth leading cause of death in the United States, where the death rate has increased to 146.2% in 2018 and 122,019 people died (Alzheimer’s Association, 2020).

There are several limitations to deal with the pathology of AD. The drugs used to cure the cognitive impairments of the brain in AD are based on the neurotransmitters or enzyme modulation with the intranasal route for their delivery to the brain (Sood et al., 2014). However, with the use of these drugs, frequent therapy failures are being reported due to their less absorption in neuronal cell membranes, instability, brain toxicity, and other pharmacokinetic and pharmacodynamic parameters (Arias, 2014; Suri et al., 2015). Engineered nanoparticles (NPs) with unique physicochemical properties and the capability to cross the blood–brain barrier (BBB) may be a promising strategy to solve these biomedical and pharmacological problems in the treatment of brain diseases like AD (Mukherjee et al., 2020). The delivery of drugs to the brain through these NPs could enhance the pharmacokinetic and pharmacodynamic profiles of the drugs with minimal toxicity (Orive et al., 2003; Gupta et al., 2019). The therapeutic potential of the drugs can be increased by employing nanotechnology-based drug delivery approaches (Nazem and Mansoori, 2008; Brambilla et al., 2011). The most essential advantage of nanomedicines for the treatment of brain diseases like AD is the controlled release of drugs at a particular site (Betancourt et al., 2009; Safari and Zarnegar, 2014). The synthesis of these NPs for drug delivery is an emerging approach, a multidisciplinary field that provides new understandings and opens avenues to manipulate materials, tissues, cells, and DNA with at least one dimension sized from 1 to 150 nm (Li S. et al., 2018; Cheng et al., 2019; Ovais et al., 2019). Nanomedicine has made considerable achievements in many fields including medicines, pharmacy, chemical/biological detection, and optics (Binda et al., 2020; Chen K. et al., 2020; Pan et al., 2020). The present study intends to provide an overview of how nanotechnology has revolutionized AD treatment/imaging and the understanding of cellular function by mainly focusing on the state-of-the-art nanomedicine-based approaches used for AD. The current key bottlenecks and future perspective in this field are also highlighted.

In this review, we depict the advances and merits of nanomedicines in the treatment of AD, believing that the emerging nanomedicines could promote the booming growth of research in this field and become clinically available for the diagnosis and monitoring of therapeutic interventions for AD and other similar central nervous system (CNS) disorders in the future.

## Pathogenesis of AD

**FIGURE 1:**
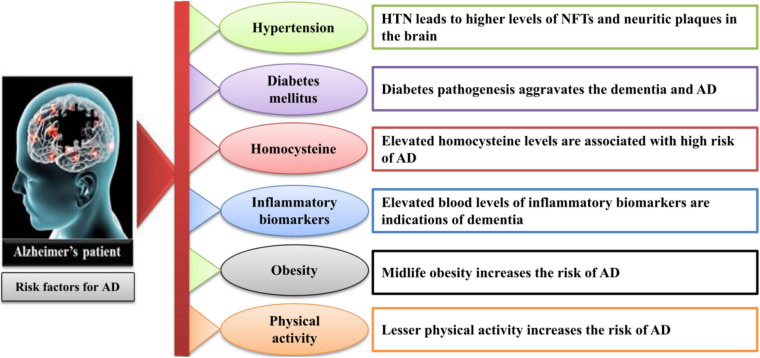
The lifestyle- and age-related factors involved in the onset of AD. AD, Alzheimer’s disease; HTN, hypertension; NFT, neurofibrillary tangles.

For a better understanding of the pathogenesis of AD, it is essential to identify the targets for direct therapy and intervention at the earlier stages when the changes are reversible. The prominent features in AD onset are the appearance of intracellular neurofibrillary tangles and extracellular amyloid plaque formation in the brain. The histopathologic traits include hippocampal neuronal loss, synaptic degeneration, and aneuploidy. Moreover, neuroinflammation, oxidative stress, microbial infection, mitochondrial dysfunction, and a compromised brain lymphatic system have also been recognized as early pathophysiological modifications in the course of AD (Swerdlow, 2007; Khoury and Grossberg, 2020). There are several physiological factors involved in the onset of AD (Harilal et al., 2019), which are summarized in Figure 1. The lifestyle- and age-related factors can aggravate the progression of AD. The following subsections describe these factors to better understand the pathogenesis of AD.

### Hypertension

The most associated, age-dependent factor that contributes to the development of AD in an aged population is hypertension. Although the mechanisms of this association are complex, it is considered that hypertension can influence AD through the association of cerebrovascular pathology (Diaz-Ruiz et al., 2009; Nehls, 2016). Hypertension–high blood pressure may also contribute to the pathology of AD as the investigation indicates that hypertension leads to the increased plaque formation in the brain (Petrovitch et al., 2000; Hoffman et al., 2009).

### Diabetes Mellitus

Recent findings have revealed the associations between high sugar level and AD development. It has been found that MA-[D-leu-4]-OB3 used for the treatment of obesity and diabetes can relieve AD pathologies (Anderson et al., 2019). About the association of AD and diabetes mellitus type 2 (T2DM), a study has reported that the depletion of caveolin-1 in T2DM can induce AD (Bonds et al., 2019). Recently, a theoretic support is depicted on the association of diabetes and AD with new targets to prevent the diabetic patients from AD (Sun et al., 2020). It has also been observed that preventive measurements against diabetes like weight–diet balance, sports, and other physical activities could relieve AD (Kang et al., 2006; Stampfer, 2006).

### Homocysteine

Homocysteine, a sulfur-containing amino acid, plays an essential role in AD-related pathologies and participates in the elevation of beta amyloid (Aβ) plaque formation (Refsum et al., 2004; Pacheco-Quinto et al., 2006). Additionally, homocysteine may contribute in raising the oxidative stress in the brain that leads to the progression of AD pathologies (Selley, 2004).

### Inflammatory Biomarkers

Inflammatory biomarkers including C-reactive proteins, interleulin-6, fibrinogen, alpha-1-antichymotrypsin, and other lipoprotein-associated phospholipase A2 have a strong association with total dementia risk (Irizarry, 2004; Mrak and Griffin, 2005). However, more findings need further evaluations.

### Obesity

As aforementioned, there is lack of literature for better evaluation of the risk factors involved in the onset of AD, but there are suggestions from different studies on the relation of midlife obesity and increased AD risk. Nevertheless, with some non-conformities, the studies based on neuroimaging, adiposity, and cognitive decline support the association of obesity and AD development (Atti et al., 2008; Beydoun et al., 2008).

### Physical Activity

Many investigations have verified the notion that the progression of AD is associated with the physical activity of a person. Healthy physical activities could retard the cognitive decline in older adults (Abbott et al., 2004; Akbaraly et al., 2009). Similarly, other studies have shown that the brain is more active during exercises such as meditation and yoga (Streeter et al., 2007; Gard et al., 2014). It has also been reported that physical activity is significantly correlated with gut microbiota that play roles in the prevention and development of AD (Schlegel et al., 2019). Another study has proved the benefit of regular exercise for AD, suggesting that exercise exerts anti-inflammatory effects and provides other numerous benefits through different pathways that might help in preventing the progression of AD (Valenzuela et al., 2020). However, the inverse correlation of physical activity and cognitive decline needs further investigations.

### Role of BBB in the Pathogenesis of AD

The human CNS is a complex system that is well distinguished by two specialized barriers in the form of cerebrospinal fluid barrier (CSFB) and BBB (Pathan et al., 2009). BBB plays a crucial role in the pathogenesis of AD. In AD, cerebrovascular dysfunction results in cognitive impairment and dementia, which may lead to cerebral amyloid angiopathy. It also mediates the accumulation of Aβ peptides in the brain. The BBB is important for the regulation of Aβ transport to brain via two primary receptors, namely, (1) receptor for advanced glycation end products (RAGE) and (2) the low density of lipoprotein receptor related protein 1 (LRP1). The faulty clearance of Aβ via the deregulated RAGE/LRP1 receptors, arterial dysfunction, and impaired angiogenesis may commence Aβ accumulation, neurovascular uncoupling, brain hypoperfusion, cerebrovascular regression, and neurovascular inflammation. Eventually, these events result in compromised BBB and subsequent neuronal and synaptic impairment (Deane and Zlokovic, 2007).

## BBB: A Limiting Factor in Drug Delivery to Brain

Blood–brain barrier plays a pivotal role in shuttling biomolecules in and out of the brain neuronal system. Therefore, understanding the structural and functional characteristics of BBB is essential for improving the drug delivery to the brain. This protective unit factor helps to prevent shuttling of molecules between blood and brain composed of vascular endothelial cell layers bound back by tight junctions and other supportive structures (Ballabh et al., 2004). The endothelial cells are surrounded by a basement membrane covered by astrocyte end-feet and continuously monitored by surveying microglial cells (Abbott et al., 2010). Cohesive domains, bound to endothelial cells, provide perseverance for the selective transport of small molecules across the BBB (Ballabh et al., 2004; Abbott et al., 2010). To meet the requirements of proteins and peptides for brain homeostasis, a controlled intracellular transport occurs via transcytosis. Depending on the nature of the molecules (hydrophilic and hydrophobic), endothelial cells with the help of various special transporting proteins could facilitate the transport. To cure brain illness like AD, different nanocarriers have been reported in preclinical studies. These carriers encapsulate the drugs against AD as cargo and cross the BBB. For a detailed view of the transcytosis and nanocarrier transport across the BBB, readers are referred to recent reviews on these topics (Villaseñor et al., 2019; Mulvihill et al., 2020).

## Nose-To-Brain Drug Delivery and Limitations

The delivery of drugs to the brain via the nasal route generally starts from the respiratory epithelium to the olfactory region with the help of the trigeminal nerve and olfactory nerve cells. This drug delivery route is supposed to transport the drug molecules to different parts of the brain including the frontal cortex, olfactory bulb, cerebrum, and brain stem (Illum, 2000; Dhuria et al., 2010). To deal with AD, the mechanism of drug transport into the brain can be classified in two ways, namely, intracellular drug transport and extracellular drug transport.

Intracellular drug delivery is the intraneuronal route of drug transport. This route of transport is relatively slow and takes around 24 h to reach from the nasal cavity to brain cells (Kristensson and Olsson, 1971). After entering the nasal cavity, exploiting the mechanism of endocytosis, the drugs could move to olfactory sensory neurons and peripheral trigeminal neurons via the olfactory and respiratory epithelium, respectively. Further from nerve cells, intracellular and transcellular transports mediate the movement of drug to different parts of the brain. The intracellular route delivers the drug to the olfactory lobe from the olfactory nerve and to the brain stem from trigeminal nerves. At the same time, the transcellular route provides the drug to the lamina propria, which further enters the brain through different ways. This type of transport facilitates the delivery of those drugs coated with lipophilic molecules that can adopt the passive diffusion or active transport/receptor-mediated transcytosis (Lochhead and Thorne, 2012). Extracellular transport facilitates the transport of hydrophilic drug substances, various proteins, and peptides. This is the sharp route of drug delivery from the nose to the brain and it is further divided into slow and fast extracellular transport.

**FIGURE 2:**
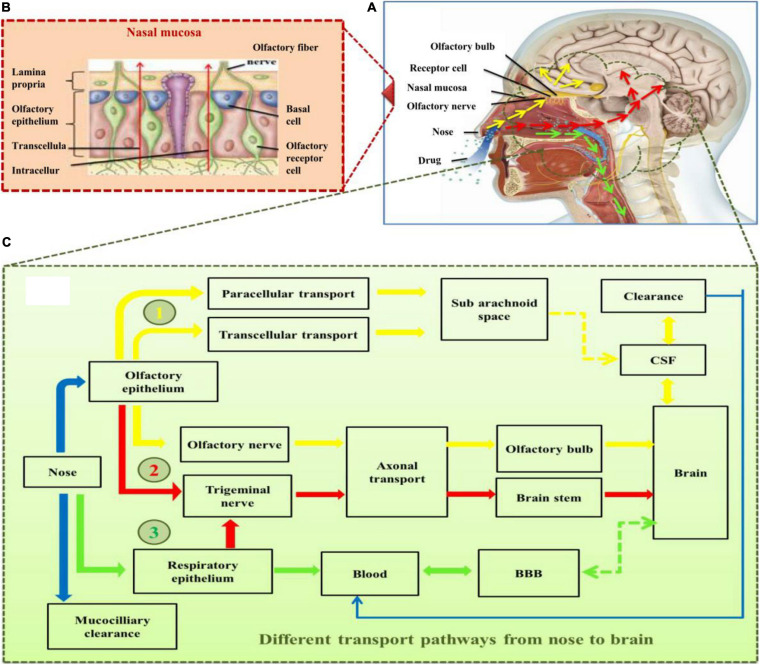
**(A)** Skull structure presenting major components of nose and brain involved in drug transport to the brain, **(B)** structural components of nasal mucosa, **(C)** the various pathways (highlighted in different colors; yellow, red, and green) for the delivery of drugs into the brain through intranasal route. Yellow arrows present the major direct pathway involving the olfactory nerve, red arrows present the pathway primarily involving the germinal nerve route, and green arrows illustrate the minor pathway involving blood–brain barrier (BBB) following the systemic absorption of the drug molecules through respiratory epithelium. BBB, blood–brain barrier; CSF, cerebrospinal fluid.

Extracellular transport of drug is the fastest route of drug delivery from the nose to the brain. In this route, drug molecules use the intercellular clefts in the olfactory and respiratory epithelium and extracellular transport along the olfactory and trigeminal neural pathway to reach the spinal fluid and brain. Once the drug reaches the lamina propria, there are different options including (i) getting into systemic circulation via being absorbed in olfactory blood vessels, (ii) it may enter nasal lymphatic vessels, (iii) or it may enter the cranial compartment associated with olfactory nerve bundles by extracellular diffusion (Lochhead and Thorne, 2012). The challenges encountered in this route are mostly associated with the physicochemical properties of drugs, including molecular weight, lipophilicity, characteristics of the drug formulation, and the presence of specific receptors on the mentioned routes (Illum, 2000). The drug in the nasal route may be eliminated by mucociliary clearance or through CSF into blood (Harush-Frenkel et al., 2008). It is also reported that the surface charge, type, and concentration of the nanomedicine carrier also influence the drug delivery to the brain (Jones, 2008). In other limitations, sometimes due to the nature of the NPs used, e.g., phospholipid complexes target inflammatory sites and the reticuloendothelial system by themselves (Khan et al., 2013). Some other functionalized NPs tend to adsorb an arbitrary biological entity and form a protein sheath, referred to as a “protein corona,” which leads to the non-targeted interaction of drugs and also random deposits/accumulation of the carrier substance in biological systems (Salvati et al., 2013; Zanganeh et al., 2016). In order to achieve a sufficient therapeutic level in the target region of human brain, there has been a continuous struggle to improve the uptake of those drug-encapsulated nanocarriers and to explore the safest transport mechanisms after absorption. Various pathways for the delivery of therapeutic moieties from the nasal route to the brain are illustrated in Figure 2.

## Nanomedicines: A Promising Approach for Drug Delivery Through BBB

**FIGURE 3:**
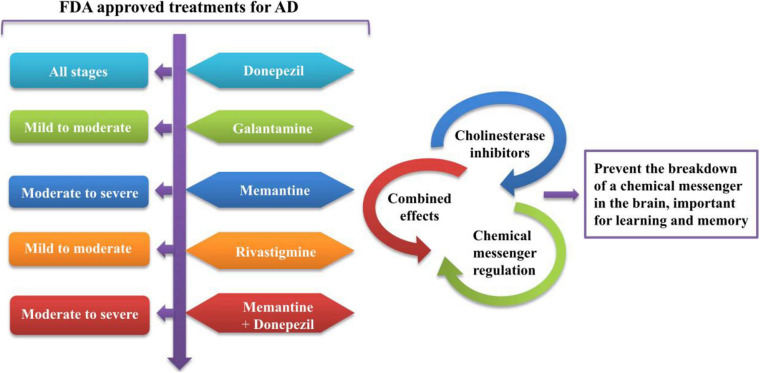
The functioning of currently FDA-approved drugs to treat the symptoms of AD. FDA, Food and Drug Administration; AD, Alzheimer’s disease.

**FIGURE 4:**
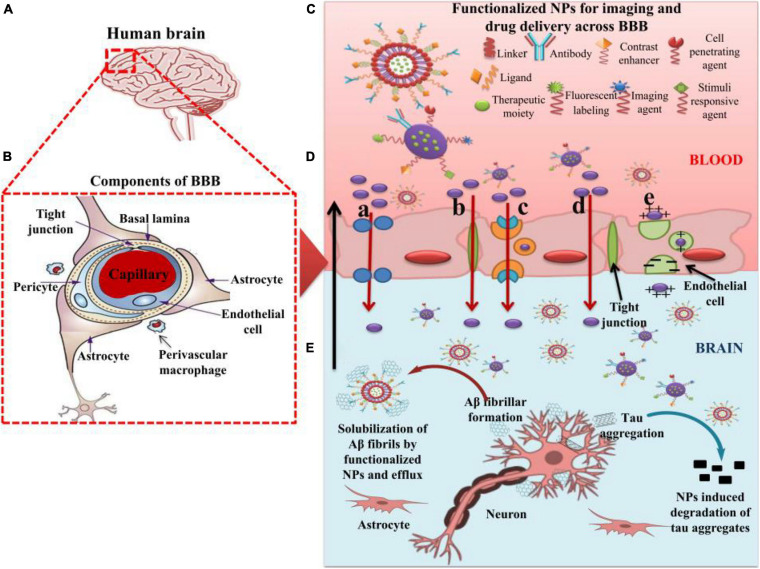
The role of nanoparticles in overcoming the BBB for efficient delivery of therapeutic moieties to treat AD. **(A)** Image of human brain. **(B)** Components of the BBB. **(C)** Functionalized nanoparticles (NPs) for imaging and targeted drug delivery to the AD brain. **(D)** Different pathways of transport **(a–e)** across BBB utilized by functionalized NPs. **(a)** Transport of NPs through cellular transport proteins; **(b)** transport of NPs through tight junctions; **(c)** transport of NPs via receptor-mediated transcytosis; **(d)** transport of NPs via transcellular pathway following diffusion, specifically adopted by gold NPs; **(e)** transport of cationic NPs and liposomes via adsorption-mediated transcytosis. **(E)** Effect of functionalized NPs in treating AD via the degradation of tau aggregates and efflux of Aβ fibrils after getting solubilized by the NPs. AD: Alzheimer’s disease; NPs: Nanoparticles; BBB: Blood–brain barrier.

For the safe and effective delivery of U.S. Food and Drug Administration (FDA)-approved (FDA, 2019), commercially available drugs, several small-sized nanocarriers have been adopted to cure brain illnesses including brain cancer and AD. These nanocarriers with specific drugs belong to nanomedicines. While there is no cure for AD, Figure 3 depicts the function of currently FDA-approved drugs to treat the symptoms of AD. The current anti-AD drugs can improve the clinical symptoms rather reversing or preventing the progression of the disease. To deliver these recommended drugs to the affected part of the AD brain, NP-functionalized nanomedicine is considered as the most useful applicable approach. Nanomedicines have a set of unique properties that enable them to deliver the anti-AD drugs at target sites in the brain. Nanomedicines have the advantages of reduced dimensions and increased biocompatibility that facilitate easy transport of therapeutic substances into the brain (Spuch et al., 2012; Fakhoury et al., 2015; Leszek et al., 2017). Small-size (approximately 100–10,000 times smaller than a human cell) nanomedicines can easily interact with the proteins and molecules on the cell surface as well as inside the cell (Kim et al., 2012). NP-functionalized nanomedicines have central core structures that ensure the encapsulation or conjugation of drugs and provide the protection and prolonged circulation in the blood (Knop et al., 2010; Li Y. et al., 2018). Nanomedicines are also specialized to target cells or even an intracellular compartment like Aβ in cells and thus can deliver the drug at a predetermined dosage directly to the pathological site (Gao and Jiang, 2006). Nanomedicines can minimize the dose and frequency and then improve patient compliance (Altinoglu and Adali, 2020). Regardless of some clinical issues, nanomedicines have potential advantages of favorability to the brain, greater stability, biocompatibility and biodegradability, protection from enzymatic degradation, increased half-life, improved bioavailability, and controlled release over other conventional ways of drug delivery to the brain to cure AD (Altinoglu and Adali, 2020). Figure 4 demonstrates how functionalized NPs have been employed to overcome the BBB, exploiting different transport pathways to achieve anti-AD effects of the delivered cargoes.

## Nanomedicines to Manage AD

Nanomaterials are being widely explored to manage the pathologies of AD. The following sections describe how nanostructure-based delivery systems are being employed for the diagnosis and treatment of AD. Table 1 presents the most recent applications of functionalized nanomaterials as carriers for delivering therapeutic moieties and imaging agents to brain for managing AD-related pathologies. Here, we present an updated view of nanomedicines as nanocarriers for delivering therapeutic moieties.

### Organic Nanostructures

#### Polymeric-Based NPs

Polymeric biodegradable NPs functionalized with PEG and antibody have been successfully designed and tested in transgenic AD mice. A recent study reveals that PEGylated NPs’ exposure can lead to the correction of memory defect and a significant reduction in Aβ soluble peptides. Thus, the designed formulation can be used to cure AD illness (Carradori et al., 2018).

To enhance the efficacy of memantine against AD, a study has been conducted in which memantine is loaded into biodegradable polymeric NPs synthesized by double emulsion method. Targeting the AD brain with memantine-loaded NPs can lead to a significant reduction in Aβ plaques and AD-associated inflammation (Sánchez-López et al., 2018). Similarly, vitamin D binding protein, a therapeutic candidate to relieve AD symptoms, has been loaded in biocompatible and degradable poly lactic-co-glycolic acid (PLGA) NPs to treat AD. It has been shown that significant reductions in Aβ accumulation, neuroinflammation, neuronal loss, and cognitive dysfunction in transgenic AD mice model have been observed (Jeon et al., 2019). Targeting the brain with zinc-loaded polymeric NPs can also lead to the decrease in amyloid plaque size and mitigate other neuronal dysfunctions in AD mice (Vilella et al., 2018). The polysaccharides in NP formulations have several advantages in biological systems, including being highly stable, being non-toxic, being biodegradable, and having a hydrophilic nature (Liu et al., 2008; Venkatesan et al., 2016; Jhaveri et al., 2021). It has been investigated that sitagliptin (SIT) [a dipeptidyl peptidase-4 (DPP-4) inhibitor]-loaded NPs show effective therapy against AD symptoms in an animal model (Wilson et al., 2020). To deliver huperzine A, an acetylcholinesterase inhibitor, the mucoadhesive and target PLGA-NPs with surface modified by lactoferrin-conjugated N-trimethylated chitosan have been adopted. The formulation has demonstrated a good sustained-release effect and target ability against AD pathologies (Meng et al., 2018). A special nanocarrier system of alginate-chitosan NPs can act as an excellent transporter to deliver the SpBMP-9, a short peptide derived from bone morphogenetic protein (BMP-9), across the BBB to AD brain. SpBMP-9 is known as a candidate drug for AD for its function in promoting the differentiation of cholinergic neurons and inhibiting GSK3β (Elnaggar et al., 2015; Beauvais et al., 2016; Lauzon et al., 2018). Similarly, with the delivery of another AD drug, Perlerin, CS-NPs have shown significant effective results with no brain toxicity and improved cognitive skills in AD Wistar rats (Hanafy et al., 2015). Trimethyl chitosan-PLGA NPs provide excellent transport of coenzyme Q10 into the brain of transgenic mice where it protects the brain from AD pathologies (Wang et al., 2010).

Curcumin is a naturally occurring antioxidant with low toxic nature and a free radical scavenger phytochemical (Kim et al., 2008; Mishra and Palanivelu, 2008; Shen and Yu, 2008). Curcumin provision to brain against tau protein aggregation in AD is considered the most attractive approach in AD treatment where it binds with tau protein-based amyloid and shows anti-amyloid properties in the micromolar concentration range (Yang et al., 2005; Garcia-Alloza et al., 2007; Mohorko et al., 2010; Re et al., 2010; Yanagisawa et al., 2010). Furthermore, there is an inhibitive effect of curcumin on the hyperphosphorylation of tau proteins (Park et al., 2008). Curcumin delivery to the brain faces the issues of poor stability and bioavailability that lead to less brain uptake (Anand et al., 2007). To deal with these issues, use of nanocarriers is preferred due to its safety, as well as higher and prolonged exposure to the brain. Various specialized NPs have been designed that encapsulate the curcumin and deliver it to the brain via transcytosis across the BBBs. Due to the friendly nature of the biological system, use of PLGA NPs is a common practice. Curcumin loaded with PLGA NPs can permeate through the BBB and reach the AD regions where it shows protective effect against the beta amyloid accumulation (Joseph et al., 2018). A stabilized and sustained curcumin delivery across the BBB is also achieved by using a nanoemulsion of red blood cell membrane-coated PLGA particles with embedded T807 molecules on the red blood cell membrane surface (T807/RPCNP) loaded with curcumin. Mutual effects of T807, 807/RPCNP exhibit strong inhibitory effects against tau-associated pathogenesis (Gao C. et al., 2020). In another study, hydroxypropyl-cyclodextrin-encapsulated curcumin complexes (CUR/HP-CD inclusion complexes) emulsion shows greater cellular uptake of curcumin across the BBB and can be considered as a better carrier system to deliver curcumin to the brain for AD therapy (Zhang et al., 2020). Furthermore, curcumin loaded with chitosan and bovine serum albumin NPs increase the drug permeation and accelerate the phagocytosis of the Aβ peptide to relieve AD symptoms (Yang R. et al., 2018). Other biological advantages of curcumin-based nanomedicines against brain diseases include its neuroprotective role by activating the transcription factor Nrf2, which is known as a master regulator of antioxidant response (Yang et al., 2009) and protects neuronal cells from dopaminergic toxicity (Szwed and Miłowska, 2012). In the Aβ-induced rat model, curcuminoids regulate the proliferation of neuronal stem cells via different kinase pathways (Ahmed et al., 2010; Liao et al., 2012). The use of curcumin for AD therapy could improve the neuronal cognition in the rat model (Xu et al., 2007; Dong et al., 2012). It plays important roles in neurogenesis, synaptogenesis, and migration of progenitor cells (Kang et al., 2006). Furthermore, curcumin-containing PLGA NPs are involved in the expression of genes that lead to neuronal cell proliferation and differentiation (Tiwari et al., 2014).

**TABLE 1:** The applications of functionalized nanomaterials for delivering therapeutic moieties and imaging agents to brain for managing AD related pathologies.

**Nanomaterials**	**TM/IG/FA**	**Delivery route**	**Particle size**	**Roles of materials in the enhancement of drug delivery**	**Effects on AD**	**References**
Biomimetic nano-system comprising of RVG29 and TPP attached RVG/TPP-MASLNs	GS as TM	IV	<140 nm	MA membranes were used as a bioactive material to camouflage SLNs in order to bypass RES to prolong the systemic circulation of the nano-system. RVG29 and TPP act as targeting moieties to facilitate the transport across BBB and subsequently, internalization into neuronal mitochondria.	Results of *in vivo* studies demonstrated that both RVG/TPP-MASLNs-GS and MASLNs-GS reached in systemic circulation in higher concentrations in comparison to the un-modified SLNs-GS and free GS solution. A remarkable neuroprotective effect with efficient BBB permeability and mitochondria targeting of the biomimetic nano-system in both Aβ-damaged HT22 neuronal cells and AD model mice was obtained.	Han et al., 2021
	RVG29 and TPP as FA					
EO-SLNs	EPO as TM	IP	219 nm	SLNs can enhance the bioavailability of EPO by overcoming the P-gp efflux and first pass effect. Additionally, changes in EPO from hydrophilic nature to lipophilic particle can improve the permeability to brain. Moreover, small size can facilitate the penetration into the target cells.	EPO-SLN prevented Aβ1-42-induced impairment of spatial recognition memory in AD model. Additionally, the EPO-SLN showed the anti-oxidant properties, prevented the Aβ plaque deposition, and decreased the ADP/ATP ratio, suggesting the suitability of the developed system for AD treatment.	Dara et al., 2019
PCL	Busulfan and Etoposide as TM	ICV	37–138 nm	Encapsulation into the self-assembly NPs and administration through ICV injection can improve NPs penetration in AD brain parenchyma and internalization in microglia cells.	Developed nano-system has demonstrated higher drug loading efficiency and controlled release of drugs in the targeted microglia cells, brain immature myeloid cells, in comparison to un-encapsulated drug, showing lesser side effects.	Peviani et al., 2019
USPIONs	Iron oxide as multi modal contrast agent	IV	12 nm	DPA-PEGylated USPIONs labeled with PH-1 and PH-2 (Phenothiazine-based small molecules), where, PEG was chosen as a linker, owing to its ability to reduce protein absorption and increase the circulation time of NPs. PH-1 and PH-2 have potential to inhibit β-amyloid aggregation and to be used as NIR imaging probes for amyloid plaques in AD.	The established nano-system has simultaneously performed *in vivo* MRI and NIR based enhanced fluorescence of Aβ plaques in the brain of double transgenic mice, prevented Aβ aggregation, disaggregated the already formed Aβ fibrils and demonstrated a protective effect against the toxicity of human neuroblastoma cells induced by Aβ_1–42_.	Cai et al., 2020
	PH-1 and PH-2 as theranostic agents					
PC-Fe_3_O_4_ and CdS-NPs	Fe_3_O_4_ and CdS as tau aggregation inhibitors	—	10–20 nm	The primary feature of the NPs developed is their complete biological synthesis using two fungal species; *Fusarium oxysporum* and *Verticillium* sp. The magnetite NPs were capped with hydrolytic proteins from fungi, while the CdS NPs were capped with four different kinds of proteins belonging to the group of sulfate-reducing enzymes. CPs avoid the aggregation of NPs.	The designed formulations have not affected the viability of neuroblastoma cells. Furthermore, PC-CdS NPs showed dual properties of disaggregation and inhibition of Tau. Hence, the NPs could be used as potent Tau aggregation inhibitors and can be subjected to several modifications for specific drug delivery owing to their very small size.	Sonawane et al., 2019
PBNPs	FAM, TM	—	50 nm	Based on florescence quenching ability of PBNPs and that DNA can adsorb on PBNPs surface via binding of phosphate skeleton in DNA to Fe^2+^/Fe^3+^, FAM-AptAβ@PBNPs-based florescent aptasensor to detect the Aβ_40_O was established.	Results indicated the sensitivity, selectivity, simplicity and applicability of the designed aptasensor for early diagnosis of AD. AD patients can be distinguished from healthy persons using this approach to detect Aβ_40_O levels in clinical samples of cerebrospinal fluid.	Chen W. et al., 2020
Lipid based NPs decorated with multi-target directed ligands	SHPs (SHP-2-Bn and SHP-2-R) as antioxidents and AChE and BChE inhibitors	Intranasal route	100 nm	Delivery system based on L-α-phosphatidylcholine and two SHP were developed as a multi-target treatment of AD. Insoluble SHP-2-Bn was chosen for modification of phospholipid membrane to prevent oxidation and membrane denaturation, whereas, water-soluble SHP-2-16 was chosen as an AChE inhibitor.	Results indicated that established nano-system has significantly reduced the scopolamine-induced AD-like dementia in rats. Moreover, the multi-target nano-system displayed the highest antioxidant activity with no toxicity and significant inhibition of brain AChE.	Burilova et al., 2020
DTNPs	Dopamine	Intra-ventricular	140 nm	Dopamine and tryptophan have been known to exhibit excellent anti-aggregation properties, neuroprotective effects, and anti-amyloid and fibril disaggregation activity, along with inherent fluorescent property. Tryptophan can cross the BBB via LAT1, thus, its presence in the nano-system can facilitate the delivery system to cross the BBB efficiently.	*In vitro* and *in vivo* investigations displayed the neuroprotective effects in neuroblastoma cells and anti-aggregation efficacy of DTNPs in both FF derived amyloid fibrils and preformed Aβ-peptide fiber, along with improved cognitive impairment in ICV-STZ induced animal model of dementia. Moreover, DTNPs also showed fluorescent properties and lighted up the cytoplasm of neuroblastoma cells illustrating their capacity to be used as an-intracellular bio-imaging agent.	Sharma et al., 2020
	Tryptophan					
Solid lipid nanoparticles SLN and NLC	Quercetin as TM	—	200 nm	Quercetin exhibits strong neuroprotective effects in AD. Lipid NPs can prevent the photodecomposition, and degradation of quercetin. NPs were decorated with transferrin to help the transport of NPs across BBB via transferrin receptors expressed on brain endothelial cells.	Permeability studies across cell monolayers displayed that NLC can effectively permeate the BBB, and amyloid-β studies showed NPs potential to inhibit fibril formation. The developed system proved to be efficient in site-specific delivery of quercetin in brain cells.	Pinheiro et al., 2020a
	Transferrin as FA					
RVG29- NPs	Quercetin as TM	—	250 nm	Lipid NPs were decorated with the RVG29 to improve the brain targeting via nicotinic acetylcholine receptors. Quercetin was encapsulated into NPs owing to its neuroprotective effects.	NPs did not show any cytotoxicity in hCMEC/D3 cell line and RVG29- NPs have increased the permeability up to 1.5 folds across the BBB in comparison with non-functionalized NPs. Finally, NPs showed the inhibition of amyloid-beta aggregation illustrating the neuroprotective potential of the formulation.	Pinheiro et al., 2020b
	RVG29 as FA					
Amyloid-β oligomer-targeted gadolinium-based NIR/MR multi-modal theranostic nanoprobe	F-SLOH as targeting and imaging agent	IV	50 nm Aβ oligomer-selective cyanine dye	F-SLOH is an Aβ oligomer-specific cyanine dye that can facilitate target specific imaging. Based on biocompatibility, tunable bio-distribution, and multimodal imaging potentials, Gd3+-based NPs have been chosen to develop multimodal targeted theranostic probe.	The nanoprobe displayed the excellent diagnostic capabilities, along with inhibitory effect on Aβ fibrillation and aggregation, and strong neuroprotection against Aβ-induced toxicity. Moreover, NPs were demonstrated to be cell membrane permeable, biocompatible, Aβ-targeted and BBB-penetrable, suggesting their potential to be used as an excellent theranostic for AD.	Gao W. et al., 2020

*IG, imaging agent; FA, functionalizing agent; TM, therapeutic moiety; GS, genistein; RVG29, rabies virus glycoprotein 29; TPP, triphenylphosphine; NPs, nanoparticles; MA, macrophage membrane; AD, Alzheimer’s disease; FAM, carboxyl fluorescein; IV, intravenous; EPO, erythropoietin; EO-SLNs, erythropoietin loaded solid lipid nanoparticles; MASLNs, macrophage membrane (MA)-coated solid lipid nanoparticles; RES, reticuloendothelial system; PCL, self-assembly poly-caprolactone; ICV, intra-cerebro-ventricular; PEG, polyethylene glycol; IP, intraperitoneal; ATP, adenosine triphosphate; ADP, adenosine diphosphate; P-gp, *P*-glycoprotein; Fe_3_O_4_, iron oxide; AChE, acetylcholinesterase; BChE, butyrylcholinesterase; LAT1, L-type amino acid transporter; NIR, near-infrared; USPIONs, ultrasmall superparamagnetic iron oxide nanoparticles; MRI, magnetic resonance imaging; PC, protein-capped; CdS, cadmium sulfide; FF, fibril formation; PBNPs, Prussian blue NPs; FAM, carboxyl fluorescein; BBB, blood brain barrier; SHP, sterically hindered phenols; DTNPs, dopamine tryptophan-nanocomposites; SLN, solid lipid nanoparticles; NLC, nanostructured lipid carriers.*

#### Nanomicellar

A nanomicellar water-soluble formulation of coenzyme Q10 (UbisolQ10) is applied to double transgenic AD mice in the form of drinking water. The results reveal that it improves the long-term memories and inhibits the levels of circulated Aβ plaques (Muthukumaran et al., 2018). It has been observed that combined micelles with Tween-80 to formulate curcumin micelles can increase the availability and efficacy of curcumin in the treatment of AD symptoms (Hagl et al., 2015). Recently, the effects of PEG ceramide nanomicelles on neuronal N2 cells have been investigated. It has been found that applied nanomicelles effectively mediate the degradation of tau proteins and induce autophagy in target cells (Gao J. et al., 2020). Another study shows a significant inhibition of the amyloidogenesis in AD mice by employing curcumin-loaded polymeric nanomicelles as a targeted therapeutic delivery system through the glycation method of bovine serum albumin in the presence of phosphate-buffered saline (Mirzaie et al., 2019).

#### Dendrimers

Dendrimers are considered as promising materials for the treatment of AD (Aliev et al., 2019). A novel finding has been achieved by coupling the lactoferrin and low-generation dendrimers for brain-targeted delivery of memantine in AD-induced mice. A recent study has reported the significant impact on the memory aspects in target mice (Gothwal et al., 2019). To increase the efficacy of drug-related CNS disorders such as AD and PAMA, dendrimers with ethylenediamine core, generation 4.0 and 4.5, are commonly used to enhance the drug solubility and bioavailabity for greater permeation across the BBB to target the damaged parts in brain (Igartúa et al., 2018). The dendrimers with poly(propylene imine) core and maltose-histidine shell (G4HisMal) have been successfully designed and could exhibit substantial improvement in AD symptoms including memory impairment (Aso et al., 2019; Igartúa et al., 2020). Co-administration of tacrine with both generation 4.0 and polyamidoamine dendrimers as nanocomposites has also been used to enhance the biocompatibility and reduce the toxicity of drugs used for the therapy of AD (Igartúa et al., 2020). Furthermore, the nanocomposites of poly(amidoamine) dendrimer and gold NPs have been used to design the disposable immunoplatforms for simultaneous determination of the biomarkers for AD (Serafín et al., 2020).

#### Nanogels

Currently, nanogels possess the ability to hold active molecules, macromolecules, and drugs together, which are considered promising drug delivery vehicles and have been exploited in many challenges associated with different kinds of pathologies including AD (Aderibigbe and Naki, 2018). A recent study reveals that the delivery of deferoxamine in the form of nanogels using the chitosan and tripolyphosphate via ionotropic method could be one of the effective therapies against AD (Ashrafi et al., 2020). Artificial chaperones in the form of polysaccharide pullulan backbones with cholesterol moieties have shown a significant effect on relieving AD pathologies by inhibiting the formation of Aβ amyloids (Ikeda et al., 2006). In a preclinical study in mice, it has been evaluated that the nose-to-brain delivery of insulin, a candidate drug for AD, can be enhanced by using nanogels as carrier (Picone et al., 2018).

### Lipid-Based NPs for AD Therapy

Many studies indicate the extraordinary importance of lipid-based nanocarriers for their usage in the drug delivery systems to cure CNS diseases like AD. Lipid NPs have remarkable potentials in delivering anti-AD drugs via nasal routes to manage AD (Akel et al., 2020).

#### Solid Lipid NPs

Solid lipid NPs are considered as excellent carriers for a-bisabolol in AD brain. The formulation has shown significant inhibitory effects against amyloid aggregation (Sathya et al., 2020). Recently, a novel approach is introduced to induce the expression of p-glycoprotein and breast cancer resistance protein transporters on brain endothelial cells via targeting the MC11 ligands. The transferrin-functionalized nanostructured lipid carriers could induce the expression of these proteins, which can be considered as a potential strategy toward AD therapy (Arduino et al., 2020). In both *in vivo* and *in vitro* experiments, the formulation of solid lipid NPs loaded with donepezil has the potential to enhance the drug delivery to the brain through the intranasal route (Yasir et al., 2018b). In another study, solid lipid NPs and donepezil formulation can be prepared by the solvent emulsification diffusion technique. The result exhibits a promising improvement in drug efficacy as compared to other formulations (Yasir et al., 2018a). Recently, the curcumin-loaded lipid-core nanocapsules have been successfully designed. The curcumin nanocapsules have shown significant neuroprotective effects against Aβ 1-42-induced behavioral and neurochemical changes in AD mice model (Yavarpour-Bali et al., 2019). Lipid carriers with curcumin nanostructures were used to treat oxidative stress parameters in AD brain to improve and recover memory conditions. These nanostructures have the potential of suppressing the hallmarks of Aβ in AD (Sadegh Malvajerd et al., 2018). Similarly, in another study, both nanostructure lipid carriers and solid lipid NPs loaded with curcumin exhibit significant neuroprotective effects against AD pathologies with greater bioavailability of curcumin to the brain (Sadegh Malvajerd et al., 2019).

#### Liposomes

The nanosized vesicular liposomes possess self-assembling and amphiphilic properties and have been extensively used as nanocarriers to deliver drugs to brain tissues (Micheli et al., 2012). Liposomes can be readily functionalized and surface modulated using several polyether, functional proteins and cell-penetrating peptides (CPPs) that aid in target-specific drug transport across the BBB (Rocha, 2013). For instance, polyethylene glycol (PEG)-coated liposomes are reported to successfully evade the opsonization of RES. In addition, glutathione-PEGylated liposomes are also reported to efficiently enhance the cellular uptake of the drug across endothelial BBB (Wong et al., 2012; Rip et al., 2014). Curcumin-loaded liposomes can significantly enhance the delivery of drugs to CNS via corresponding receptors on BBB cells (Mourtas et al., 2014; Lajoie and Shusta, 2015). To deliver apolipoprotein E (ApoE_2_) in AD brain, the liposome carrier system altered with surface containing mannose ligand and CPPs have been applied. The results indicate that functionalized liposomes are safe and compitable and can deliver substantial concentration of genes to the target tissues in AD therapy (Arora et al., 2020). Due to the protective effects for hippocampus neurons and anti-Aβ properties, osthole (Ost) is considered as an anti-AD compound. An Ost-liposomes carrier system has been developed for its bioavailability and the exposure to target sites in the AD mice brain (Kong et al., 2020).

#### Niosomes

Administration of a niosome–lipid nanocarrier system loaded with artemisia-absinthium against amyloid aggregation shows significant effects on AD pathologies. Thus, the formulation can be used to preclude the development of amyloid for AD therapy (Ansari and Eslami, 2020). In order to enhance the drug exposure to AD brain via the intranasal route, pentamide-loaded chitosan glutamate-coated niosomes have been developed. The results exhibit a significant increase in pentamide efficacy across the BBB (Rinaldi et al., 2018). Low blood level of folates is considered as the primary cause of AD. To deal with the hindrance of folic acid transport across the BBB, formulation with different concentrations of folic acid-niosomes has been prepared. It has been found that niosomes with span 60 and cholesterol in the ratio of 1:1 (50 mg:50 mg) show higher entrapment efficacy with more exposure to the affected parts of the brain (Ravouru et al., 2013). Rivastigmine is an acetylcholine esterase inhibitor and can improve brain functions in CNS disorders like AD. A niosome formulation is prepared using sorbitan esters and cholesterol by film hydration technique. The formulation exhibits amazing results in improving drug efficacy to target brain tissues (Estabragh et al., 2018).

#### Nanoemulsion

Nanoemulsion formulations maximize the efficacy of anti-AD drugs and make them specific against specific target sites in the brain (Nirale et al., 2020). A nanoemulsion using homogenization and ultrasonication has been used to load memantine for intranasal delivery to bypass the BBB for AD therapy. Both *in vivo* and *in vitro* experiments reveal the promising effects of emulsion against AD pathologies (Kaur et al., 2020). In order to improve the clinical usage with enhanced efficacy, naringerin nanoemulsion is further prepared. The result reveals that nanoemulsion from naringerin could be a potential approach to overcome Aβ neurotoxicity and amyloidogenesis (Md et al., 2018).

#### Cubosomes

Cubosomes are another lipid-based NPs that may have the potential biomedical application for drug delivery to the brain (Gaballa et al., 2020). The results of donepezil-HCL delivery through cubosomal mucoadhesive *in situ* nasal gel show that formulated gel could be considered as a promising carrier for drug delivery to target the affected parts of the brain (Patil et al., 2019).

#### Amylolipid Nanovesicles

To achieve the maximum drug concentration across the BBB for rapid and greater impact of drugs on brain cells, a novel lipid-based nanocarrier system has been developed. It is a self-assembled lipid-modified starch hybrid system. A study demonstrates that intranasal administration of curcumin loaded in amylolipid nanovesicles has greater tendency to cross the BBB and shows significant effect against AD pathologies. Thus, the findings prove that this carrier system is a promising carrier for drug delivery to AD brain tissues (Sintov, 2020).

### Metallic NPs

In nanomedicine-based approaches to AD, the use of metallic NPs is considered a potential research area for targeted drug delivery across the BBB. Due to the utilization of chemistry-based techniques in their synthesis, metallic NPs have some limitations, but some of the metallic NPs like cerium, selenium, gold, and iron are known to exhibit significant anti-AD properties. Currently, researchers are oriented toward the use of green chemistry-based approaches for designing biologically friendly NPs.

#### Selenium NPs

As aforementioned, reducing ROS level in the brain is a key strategy to relieve AD. There are many trace elements such as selenium (II), sodium selenite (VI), and sodium selenite (IV) known as active ROS inhibitors. Being important micronutrients of the human body and gifted with biomedical application of selenium nanoformulation, selenium- and selenite-containing NPs play roles in lowering the oxidative stress and inhibiting the cytotoxicity of cells. Therefore, they have the potential to be used in curing neurodegenerative diseases like AD (Fernandes and Gandin, 2015; Rajeshkumar et al., 2019). It has been found that the modified selenium NPs with sialic acid can cross the BBB and their exposure can inhibit the Aβ aggregation reactions (Yin et al., 2015). Similarly, sialic acid-modified selenium NPs coated with high BBB permeability peptide-B6 and epigallocation-3gallate (EGCG) could inhibit the Aβ aggregation (Zhang et al., 2014). In a transgenic AD mouse model, a novel modified nanoformulation of selenium NPs encapsulated into PLGA nanospheres with curcumin has exhibited strong inhibitory effects against Aβ aggregation, which can be considered as a valued delivery system for targeted drug delivery in the treatment of AD (Huo et al., 2019).

#### Cerium NPs

Cerium oxide NPs could protect vital neuronal function against high ROS levels in an AD patient. It has been evaluated that CeONPs have no negative effects and are extremely useful for the treatment of AD. The success of AD treatment with ceria can be attributed to greater uptake across the BBB and no unwanted accumulation in other biological sites (Rzigalinski et al., 2017; Wahle et al., 2020). In a preclinical AD mouse model, ceria NPs coupling with triphenylphosphonium (TPP) localize in the mitochondria and prevent neuronal death (Kwon et al., 2016). In another study, γ-FeO3/CeOx@PEG2,000 NPs could effectively scavenge radicals and decrease the oxidative stress (Horák et al., 2020).

#### Gold NPs

Gold NPs play important roles in drug delivery across the BBB to the brain for the treatment of neurodegenerative diseases. There are various AuNP formulations that are used for the diagnostic and therapeutic strategies in the treatment of AD (Sivanesan and Rajeshkumar, 2019). In the AD mice model, D-glutathione-stabilized gold NPs can cross the BBB following intravenous administration and show strong inhibitory effects against Aβ42 aggregation with no neurotoxicity (Hou et al., 2020). Another study reveals that treatment with gold NP formulation via intrahippocampal and intraperitoneal injections could improve the acquisition and retention of spatial learning and memory (Sanati et al., 2019). To enhance the neuroprotective efficacy of dietary polyphenolic compounds like anthocyanin, conjugation of anthocyanin with gold NPs has been developed. It has been demonstrated that treatment with anthocyanin-loaded PEG-AuNPs in an amyloid beta mouse model of AD is a promising strategy to prevent the age-associated neurodegenerative disease (Ali et al., 2017). A recent study indicates that administration of maize tetrapeptide-anchored gold NPs can improve central cholinergic system function and reduce the level of acetylcholinesterase, suggesting that novel tetrapeptide can be used as a neuroprotective agent to prevent AD (Zhang et al., 2021). Another study has reported that treatment with AuNPs in AD animals has significantly reversed the symptoms of AD by reducing neuroinflammation and modulating mitochondrial functions (Dos Santos Tramontin et al., 2020).

#### Iron NPs

Iron oxide NPs have been widely used in biomedical studies. It has been investigated that ultrasmall superparamagnetic iron oxide NPs coupled with phenothiazine-based near-infrared (NIR) fluorescent dye can act as novel theranostic agents for AD. The particles have the ability to perform NIR fluorescence and magnetic resonance imaging of Aβ plaques and prevent their aggregation in the brain of AD mice (Cai et al., 2020). In addition, treatment with protein-capped (PC) Fe_3_O_4_ and PC-cadmium NPs can act as potent tau aggregation inhibitors in AD cells, which may provide a novel strategy to design anti-tau aggregation drugs for AD patients (Sonawane et al., 2019). Furthermore, the iron oxide NP formulations may possess potential applications in the diagnosis and treatment of neurodegenerative diseases such as AD (Luo et al., 2020).

### NP-Chelation-Based AD Therapy

A major pathology in AD is neuronal degeneration, and it has been found that oxidative stress is one of the leading risk factors that initiate and promote neurodegeneration. Compared to normal brain, AD brain shows the dysregulation of metal level, such as iron, aluminum, zinc, and copper, which may facilitate oxidative stress, toxic radical formation, and disruption in DNA functioning, and mediate the onset of AD symptoms (Lovell et al., 1998).

To deal with the metal accumulation and their resultant oxidative stress in the brain, nanotechnology contributes effectively to the form of chelation therapy in the inhibition of oxidative stress. To reduce the levels of corresponding metals in AD brain, NPs of Fe and Cu metals in the form of chelators have been employed. These chelators are designed to be safe in delivery and have minimal neurotoxicity to healthy brain tissues (Mandel et al., 2007). Chelation therapy shows significant effects on the solubilization of Aβ plaques in AD brain. In metal chelation, copper is the most vital trace element and 54 copper binding proteins have been found in human proteomes (Liu et al., 2009b; Blockhuys et al., 2017). It has been found that copper ions are involved in the modulation of the expression of amyloid precursor proteins, and their chelators can reduce Aβ accumulation up to 50% in AD transgenic mice. The Cu-conjugated NP formulation in the form of chelator clioquinol (CQ) has the potential to reverse the metal precipitation of amyloid protein in AD patients (Barnham and Bush, 2008). NP–chelator conjugates exhibit inhibitory effects against Aβ aggregation and protect neurons from neurotoxicity without affecting their proliferation (Liu et al., 2009b). For better intake and higher concentration, NP–iron chelator conjugates are designed with the coating of polysorbate 80 that mimic the low-density lipid (LDL) receptors on brain cells and facilitate the entry across BBB (Cherny et al., 2001; Kreuter, 2001; Kreuter et al., 2002; Cui et al., 2005).

In *in vitro* experiments, 8-hydroxyquinoline derivatives especially compound-5b chelation have shown significant inhibitory effects against the self-induced Aβ aggregation in AD. Furthermore, they exhibit no toxic effects and possess excellent penetrative tendency across the BBB (Yang X. et al., 2018). Similarly, xanthone derivatives in the chelation form also exhibit a selective inhibitory effect against a neuronal enzyme, namely, acetylcholinesterase. Based on the inhibition of acetylcholine and antioxidant activity, these derivatives have the potential to be used in the treatment of AD (Kou et al., 2020). In another study, deferasirox and tacrine chelators are designed, and their supportive roles in the treatment of AD are further evaluated. The compounds have good multifunctional activities in the inhibition of acetylcholinesterase (Wang et al., 2019). Nano-N2PY, a NP–chelator conjugate, plays a significant role in protecting cortical neurons from Aβ-related toxicity (Liu et al., 2009a). Similarly, other metal chelators such as ethylene diamine tetra acetic acid (EDTA), iodochlorhydroxyquin (clioquinol), and deferoxamine are being used against AD pathologies and exhibit promising results in AD treatment (Ritchie et al., 2003).

### Nanomedicine–Theranostics Formulations

#### Gold (Au) NPs

The mechanism involved in the treatment of Aβ fibrils with gold-containing NPs (AuNPs) is the same as treating cancer cells with metallic NPs (Kabanov and Gendelman, 2007). The molecular docking, system biology, and time course simulation analysis confirm and validate the synergistic role of AuNPs in inhibiting Aβ formation in the brain (Kabanov and Gendelman, 2007). AuNPs play a promising role in diagnosing the AD both in bare form and in conjugation with other compounds. Based on the antioxidant and anti-inflammatory characters, AuNP exposure can relieve the brain damage in the AD model (Kaushik et al., 2019). AuNPs in combination with reduced graphene or coated with anti-tau antibodies can act as neuro-probes and detect the Tau-441 target proteins both in serum fluid and cerebrospinal fluid (Neely et al., 2009; Karaboga and Sezgintürk, 2020). AuNPs conjugated with Co^2+^ can be used to investigate the Aβ peptides’ aggregation kinetics and their self-assembly stages in MRI images (Sparks, 2008). In the diagnosis of AD pathologies, AuNPs follow the biosensory strategies. For example, AuNPs are used in the development of an electrochemical immunosensor to detect the tau proteins (Razzino et al., 2020). Furthermore, it has been found that chiral recognition of stable AuNPs could enhance their ability to prevent Aβ aggregation (Hou et al., 2020).

#### Protein-Coated NPs

In biomedical sciences, the usage of protein-coated NPs in multifunctional therapeutic approaches has great importance in the treatment of AD (Hong et al., 2020). It has been shown that serum albumin (SA)-NP formulation increases the efficacy of R-flurbiprofen (an anti-AD drug) in reducing Aβ peptide toxicity in the brain (Wong and Ho, 2018). Similar to R-flurbiprofen, SA-NPs are also used in the transport of tacrine and can stabilize the bioavailability with minimum hepatotoxicity (Luppi et al., 2011). In addition, the NPs coupled with BSA and sialic acid have been used to detect the early Aβ formation in the onset of AD (Zhao et al., 2015). Furthermore, delivered protein-based NPs serve as contrast agents in the brain and facilitate in providing better imaging to study the Aβ plaques (Zhao et al., 2015).

#### Antibody (Ab)-Decorated NPs

To cure AD, delivering immunotherapy doses against amyloid bodies imposes serious side effects in the form of meningoencephalitis (Gelinas et al., 2004; Moretto et al., 2007). To minimize the side effects of immunotherapy, the usage of NPs coated with antibodies for specific target proteins can be the best alternative to detect and dissolve the protein aggregations in brain cells. Using the secondary ion mass spectrometry, antibodies coated with metal oxide NPs are adopted for the imaging of AD-associated proteins in the brain (Moon et al., 2020). Chitosan-based smart nano-vehicles coated with modified Ab fragments have been used to target the Aβ amyloids in AD cells. For greater uptakes across BBB and better diagnostic approach, NP-Ab formulation is coupled with contrast agents such as FITC and Alexa Fluor (Agyare et al., 2008). Superparamagnetic iron oxide NPs conjugated with Aβ oligomer-specific scFv-AbW20 and class A scavenger receptor activator XD4 (W20/XD4-SPIONs) exhibit promising results in the therapeutic benefits for AD (Liu et al., 2020a). In another study, multifunctional superparamagnetic iron oxide NPs conjugated with Aβ oligomer-specific scFv-antibody and class A scavenger receptor activator show promising early diagnostic potential for AD (Liu et al., 2020b). PEG-NPs coated with specific Abs are used to degrade Aβ-42 (Gao W. et al., 2020). Decorating the PLGA NPs surface with 83-14 monoclonal Ab could effectively reduce the neurotoxicity induced by Aβ fibrils in AD brain (Kuo and Tsai, 2018).

## Conclusion Remarks and Future Perspective

This review has provided an outlook of the recent advances in nanomedicines employed to cure AD. Considering the ultimate goals of nanomedicines, much advancement will be achieved in the treatment of AD. The nanomedicines have tailored and transformed both diagnostic and therapeutic approaches for AD. For the promising future of nanomedicines used in AD, we suggest the revision of the current practices to consider the neglected factors at the nano–bio interface in order to minimize the risk of misinterpretations of the outcomes. Furthermore, the adoption of multipurpose NPs with multi-therapeutic capacities (e.g., delivering a variety of therapeutic moieties to manage inflammation, tau phosphorylation, oxidative stress, and mitochondrial dysfunctionality) is also recommended. Moreover, the challenges in the large-scale production of reproducible NPs need to be addressed. Considering the key targets of the current drugs involving tau proteins, neuroinflammation, and Aβ proteins, there is an urgent need to develop the drugs with new targets that can not only treat the symptoms but also prevent the progression of the disease at an early stage, which can ultimately lead to a better quality of life.

## Author Contributions

NK, D-DW, and X-YJ: conceptualization. NK, MM, and EN: data curation. X-YJ and D-DW: funding acquisition. NK, MM, EN, UZ, MK, SK, Y-KZ, E-SJ, MZ, S-FD, and J-SW: writing—original draft. D-DW and X-YJ: visualization and supervision. NK, D-DW, and X-YJ: editing. All authors: contributed to the article and approved the submitted version.

## Conflict of Interest

The authors declare that the research was conducted in the absence of any commercial or financial relationships that could be construed as a potential conflict of interest.

## Abbreviations

AD: Alzheimer’s disease
BBB: blood–brain barrier
CNS: central nervous system
NPs: nanoparticles
T2DM: diabetes mellitus type 2
RAGE: receptor for advanced glycation end products
LRP1: lipoprotein receptor related protein 1
JAMs: junctional adhesion molecules
FDA: Food and Drug Administration
PLGA-NPs: polylactide-co-glycolide-nanoparticles
PBCA: polybutyl cyanoacrylate
TPP: triphenylphosphonium
EGCG: epigallocatechin-3-gallate
TAT: *trans*-activating transduction
PEG: polyethylene glycol
RES: reticuloendothelial system
